# Occurrence of traumatic dental injuries among preschool children with Autism Spectrum Disorder

**DOI:** 10.12669/pjms.344.15681

**Published:** 2018

**Authors:** Fares S. Al-Sehaibany

**Affiliations:** 1 Fares S. Al-Sehaibany, BDS, DMSc. Associate Professor, Department of Pediatric Dentistry and Orthodontics, College of Dentistry, King Saud University, Riyadh, Saudi Arabia

**Keywords:** Autism spectrum disorder, Preschool children, Traumatic dental injuries

## Abstract

**Objective:**

To determine the occurrence of traumatic dental injuries (TDIs) among Saudi preschool children with autism spectrum disorder (ASD) and compare it with Saudi preschool children without ASD.

**Methods:**

This study was conducted on a randomly selected sample of three to five year old Saudi preschool children in Riyadh, Saudi Arabia. The sample consisted of two groups; a study group (SG) of 257 ASD children, and a control group (CG) of age- and gender-matched 257 children without ASD. Clinical examinations were performed at selected ASD centers and kindergartens to determine the occurrence of TDIs based on modified World Health Organization (WHO) classification system.

**Results:**

Out of 514 children examined, 108 (21.0%) had suffered TDIs. The prevalence of TDIs was significantly higher in the SG (25.7%) children than the CG (16.3%) [*p*=0.012]. The primary maxillary central incisors were the most commonly affected teeth in both the groups; significantly more in CG (72.9%) than SG (50.1%) [p=0.017]. Enamel fracture was the most frequent type of TDI in both the groups; significantly more in CG (29.2%) than SG (21.1%) [*p*=0.032]. Luxation injuries and tooth avulsions were significantly higher in the SG than the CG (*p*=0.028).

**Conclusions:**

The occurrence of TDIs was higher in Saudi preschool children with ASD than in non-ASD children.

## INTRODUCTION

Autism spectrum disorder (ASD) is a neuro developmental disorder that is mainly caused by genetic or neurobiological factors, and represents a wide range of intellectual deficits associated with communication and repetitive behavioral patterns.^1^,^2^ An epidemiological review on autism in Arab Gulf countries showed a prevalence of ASD ranging from 1.4 to 29 per 10,000 individuals; indicating that ASD is a serious health concern.^3^ The prevalence of ASD in Saudi Arabia was reported as 18 per 10,000 live births.^4^

Children with ASD demonstrate a variety of atypical behaviors, including peculiar mannerism, obsessions, compulsions, unusual attachments to objects, stereotype and self-injurious behaviors.^1^ Compared to non-ASD children, preschool children with ASD exhibit higher prevalence of oral habits such as bruxism, object biting, thumb sucking and tongue biting.^5^ As a result of certain behavioral aspects and oral habits, ASD children are particularly vulnerable to traumatic dental injuries (TDIs) caused by accidents or self-inflicted injuries. Risk factors for the occurrence of TDIs include demographic, socioeconomic, behavioral, anthropometric, and oral factors.^6^ The high frequency of TDIs and their negative consequences in ASD children suggest that special attention should be devoted to such injuries.^7^

Management of TDIs poses a major challenge in ASD children because these children are often unable to cooperate during dental care.^8^ Therefore, prevention of TDIs is of utmost importance in ASD children. It is essential to gauge the magnitude of TDIs in these children before designing specific preventive measures. However, there is a lack of information about TDIs in ASD children. The purpose of the present study was to determine the occurrence of TDIs among Saudi preschool children with ASD and compare it with Saudi preschool children without ASD.

## METHODS

This was across-sectional study conducted at selected ASD centers and kindergartens in Riyadh, Saudi Arabia. The study protocol and consent form were approved by the Research and Ethical Committee for Human Studies at the College of Dentistry Research Center in King Saud University, Saudi Arabia.

A stratified cluster random sampling was used to select the sample for the study group (SG) and age- and gender-matched control group (CG) children. The SG was recruited from three ASD centers randomly selected from a list of ASD centers provided by the Saudi Ministry of Education. Similarly, three kindergartens were randomly selected from a list of kindergartens provided by the same Ministry. Letters explaining the nature of the study and informed consent forms were sent to the parents of the SG and CG children through heads of the selected centers and kindergartens.

Clinical examination of the SG and CG children was performed while the child was seated on a regular chair. All examinations were performed by one experienced and calibrated examiner. Intra-examiner reliability was established via the re-examination of 30 children (15 SG children and 15 CG children) at two visits one week apart (kappa=0.88). The Federation Dentaire International (FDI) two-digit system^9^ was used for tooth numbering (Appendix-1). The modified WHO classification system^10^ was utilized to classify TDIs in the primary anterior teeth (incisors and canines) in both the SG and CG.

**Table:** Appendix 1

Tooth number*	Description
51	Primary maxillary right central incisor
52	Primary maxillary right lateral incisor
53	Primary maxillary right canine
61	Primary maxillary left central incisor
62	Primary maxillary left lateral incisor
63	Primary maxillary left canine
71	Primary mandibular left central incisor
72	Primary mandibular left lateral incisor
73	Primary mandibular left canine
81	Primary mandibular right central incisor
82	Primary mandibular right lateral incisor
83	Primary mandibular right canine

* Federation Dentaire Internationale (FDI) two-digit system.

**The following information was recorded for each participant:**

1. Age (in years), and gender.
2. Age (in years) at the time of suffering TDI.
3. Tooth type and number of injured teeth.
4. **Type of TDI:** Hard dental tissue and pulp injuries (enamel fracture, enamel/dentin fracture, enamel/dentin/pulp fracture, root fracture, crown/root fracture, and crown/root/pulp fracture); periodontal tissue injuries (subluxation, intrusive luxation, extrusive luxation, lateral luxation and avulsion).

**The following inclusion criteria were utilized:**

1. No missing tooth due to reasons other than TDIs.
2. No structural loss of one or more teeth due to dental caries.

### Statistical analysis

Statistical Package for the Social Sciences (SPSS, version 20, Chicago, IL, USA) was used to enter and analyze data obtained in this study. Data analysis included descriptive statistics and generation of frequency distributions. Independent t-test was used to compare the means values. Chi-square test was used to determine any significant relationship between categorical variables. The significance level was set at *p*<0.05.

## RESULTS

The study sample consisted of a total of 514 Saudi preschool children, 257 each in SG and CG. The age in both the groups ranged from 3-5 years with a mean age of 4.2±0.5 years for the SG and 4.0±0.7 years for the CG. The two groups had the same distribution in terms of gender, with male to female ratio of 2.3:1.

Out of 514 children examined, a total of 124 TDIs were observed in 108 children. In the SG, 66 (25.7%) children had TDIs; 56 (84.8%) had injury in one tooth and 10 (15.2%) in two teeth. In the CG, 42 (16.3%) children had TDIs; 36 (85.7%) in one tooth and 6 (14.3%) in two teeth. There were no children observed with injuries in more than two teeth. The prevalence of TDIs was significantly higher among the SG (25.7%) than the CG (16.3%) [*p*=0.012]. With respect to history of TDIs, there was no significant between-group difference in the mean age at which a TDI occurred (*p*=0.590) as shown in Table-I.

**Table I:** Prevalence of TDIs in the SG and the CG.

	Group Total N=514		p-value

	SG	CG	
Number of children	257	257	
Age in years (Mean & SD)	4.2±0.5	4.0±0.7	0.681
Children with TDIs (%)	66 (25.7)	42 (16.3)	0.012
Age in years at time of TDI (mean & SD)	3.7±0.8	3.5±0.9	0.590

In both the groups, the most frequently affected teeth were primary maxillary central incisors (teeth # 51 and 61); significantly more so in CG (72.9%) than SG (50.1%) [*p*=0.017]. In the SG, TDIs were observed in all maxillary and mandibular primary incisors and canines, while there were no TDIs observed in maxillary and mandibular primary canines of CG children. The distribution of affected teeth differed significantly between the SG and the CG (*p*=0.017) [Table-II].

**Table II:** Frequency distribution of TDIs in the SG and the CG according to tooth type.

Tooth number*	Group		p-value
	SG N (%)	CG N (%)	
51	21 (27.7)	19 (39.6)	0.017
52	11 (14.6)	5 (10.4)	
53	1 (1.3)	0 (0.0)	
61	17 (22.4)	16 (33.3)	
62	10 (13.2)	4 (8.3)	
63	1 (1.3)	0 (0.0)	
71	3 (3.9)	1 (2.1)	
72	3 (3.9)	1 (2.1)	
73	1 (1.3)	0 (0.0)	
81	4 (5.2)	1 (2.1)	
82	3 (3.9)	1 (2.1)	
83	1 (1.3)	0 (0.0)	

Total N (%)	76 (100)	48 (100)	

* Federation Dentaire Internationale (FDI) two-digit system

The types of TDIs observed ranged from enamel fracture to tooth avulsion as shown in Table-III. Overall, the most common type of TDI was enamel fracture, which was significantly higher in the CG (29.2%) than the SG (21.1%) [*p*=0.032]. There were no root fractures, crown/root fractures, or crown/root/pulp fractures observed in SG and CG. The prevalence of luxation injuries (subluxation, intrusive luxation and lateral luxation) and tooth avulsions were significantly higher in the SG (42.2% and 10.5%, respectively) than the CG (25.0% and 4.2%, respectively) [*p*=0.028]. There were no extrusive luxation injuries observed in SG and CG.

**Table III:** Frequency distribution of TDIs in the SG and the CG according to type of TDI.

Type of TDI	Group		Total N (%)	p-value

	SG N (%)	CG N (%)		
*I. Hard dental tissue and pulp injuries:*				0.032
Enamel fracture	16 (21.1)	14 (29.2)	30 (24.2)	
Enamel/dentin fracture	12 (15.7)	11 (23.0)	23 (18.5)	
Enamel/dentin/pulp fracture	8 (10.5)	9 (18.6)	17 (13.7)	
*II. Periodontal tissue injuries:*				0.028
Subluxation	11 (14.6)	4 (8.3)	15 (12.1)	
Intrusive luxation	6 (7.9)	2 (4.2)	8 (6.5)	
Lateral luxation	15 (19.7)	6 (12.5)	21 (16.9)	
Avulsion	8 (10.5)	2 (4.2)	10 (8.1)	

Total N (%)	76 (100)	48 (100)	124 (100)	

## DISCUSSION

This study provides important baseline information on occurrence of TDIs in Saudi preschool children with ASD. The major finding was that TDIs were more prevalent in the ASD children than their healthy counterparts. However, some limitations should be considered before interpreting the findings of this study. The present study did not assess possible causes of the TDIs. This study was cross-sectional in nature. It would also be interesting to perform a longitudinal study to assess whether TDIs to the primary dentition could later affect the permanent dentition in this high-risk population.

The findings of this study were consistent with those of a Brazilian study^11^ that reported TDIs prevalence of 24.6% among ASD children. Another Brazilian study^12^ reported high TDIs prevalence among ASD group (39.3%) than in CG (26.2%); in agreement with the findings of the present study. A Turkish study^13^ also reported higher TDIs prevalence in ASD than non-ASD children (23.0% and 15.0%, respectively). All of these studies^11^-^13^ included adolescents/young adults; in contrast to the current study that assessed only preschool children.

Generally, cognitive developmental delay and impaired motor coordination are the main reasons why children with special needs would be more prone to TDIs than healthy children.^11^-^14^ In the present study, both in ASD and non-ASD groups, maxillary central incisors were the most commonly affected teeth; and enamel fractures were the most frequent type of TDI. These results corroborate with the findings of previous studies in children with ASD^11^-^13^ and healthy preschool children.^15^,^16^ One possible explanation of this common finding is that these teeth are in anteriorly exposed position in dental arch. The present study showed that the occurrence of TDIs in the CG was limited to the incisors in both arches. However, in the SG a broader occurrence TDIs was observed in which the canines in both arches also suffered TDIs. The difference could be attributed to a higher prevalence of certain oral habits such as object biting and thumb sucking in ASD preschool children.^5^ These oral habits may alter the occlusion and facial pattern of the ASD children and lead to anterior open bite and increased overjet; making all the anterior teeth more prone to TDIs.^6^

In this study, luxation injuries and tooth avulsions were more prevalent in the SG than the CG, a finding that underscores the need for additional investigations. Various studies have related occurrence of TDIs with particular behavior disorders.^6^ A high prevalence of self-injury has been reported in ASD children^17^, including deliberate destruction of body tissues especially orofacial structures. This behavior is often rhythmic and repetitive and can range from mild head rubbing to sever head banging.^18^ This possibly explains the higher severity of TDIs in the SG than the CG. Given that TDIs are often encountered in clinical practice, the investigation of TDIs in ASD children is pertinent to the implementation of preventive and interceptive treatment strategies.^19^

Both TDIs and ASD have complex etiologies. According to Andreasen et al.^10^, the prevalence of TDIs vary greatly in different populations; a finding that emphasizes the effects of risk factors such as socioeconomic, behavioral, environmental and cultural factors. It also points towards lack of standardization in classification of TDIs. It is important to identify children at a greater risk of TDIs through observation of behavioral risk factors. This study has shown that children with ASD are more prone to TDIs and there is an urgent need of preventive strategies to be implemented at an early age in ASD children.

## CONCLUSIONS

The prevalence of TDIs was higher in Saudi ASD preschool children as compared to non-ASD children. The high prevalence of TDIs among the ASD children warrants special attention to the prevention and management of TDIs in the ASD children.
